# Patient adherence in orthodontics: a scoping review

**DOI:** 10.1038/s41405-024-00235-2

**Published:** 2024-07-16

**Authors:** Ronald Max van der Bie, Annemieke Bos, Jan Joseph Mathieu Bruers, Ronald Edwin Gaston Jonkman

**Affiliations:** 1 Department of Orthodontics, Academic Centre for Dentistry Amsterdam (ACTA), University of Amsterdam (UvA) and Vrije Universiteit (VU), Amsterdam, The Netherlands; 2 Department of Oral Public Health, Academic Centre for Dentistry Amsterdam (ACTA), University of Amsterdam (UvA) and Vrije Universiteit (VU), Amsterdam, The Netherlands

**Keywords:** Orthodontics, Dentistry

## Abstract

**Background:**

Patient adherence is a key factor in achieving orthodontic treatment success. Despite an evolution in orthodontic healthcare, no recent comprehensive reviews are available on patient adherence in orthodontics. This scoping review provides an evidence-based overview of the literature available on multiple aspects of patient adherence in orthodontics, during both active treatment as well as during the retention phase. Knowledge gaps identified in the literature are listed.

**Methods:**

The protocol for this scoping review was registered in the Open Science Framework (10.17605/OSF.IO/EC6QD). Electronic databases and reference lists of relevant studies were searched up to 9 February 2023. The inclusion criteria were studies investigating any form of patient adherence in orthodontics published in English from 2006 onwards. The exclusion criteria were studies investigating adherence in the following patients: those with an intellectual or physical disability that could affect their ability to coincide with their therapist’s recommendations and advice, those with oral cleft and craniofacial conditions, and those treated solely for obstructive sleep apnoea. Non-peer-reviewed studies and case reports were also excluded.

**Results:**

A total of 3284 articles were identified, 60 of which met the criteria for final inclusion.

**Conclusions:**

There is no conclusive evidence on which factors have a significant impact on patient adherence and how patient adherence can be promoted. The degree of patient adherence is generally not compared to achieved treatment results or stability of treatment results, making it difficult to provide clear statements about the impact of the degree of adherence on desired treatment results or orthodontic stability.

## Introduction

Patient adherence is a key factor in achieving orthodontic treatment success [1–3]. Poor patient adherence during and after active treatment may result in less satisfactory treatment outcomes, deleterious effects, longer duration of orthodontic treatment, and relapse after treatment [1, 3]. Although patient adherence in general healthcare is usually defined as ‘the extent to which a person’s behaviour coincides with medical or health advice’ [4], there is no precise definition of patient adherence in orthodontics. Adherence in orthodontics is generally considered in terms of wearing appliances as instructed, keeping appointments and maintaining sufficient oral hygiene [3].

There are no recent comprehensive reviews available on patient adherence in orthodontics, only reviews on specific topics of adherence, even though there has been an evolution in orthodontic healthcare. The demand for orthodontic treatment is substantial and there has been an increase in the number of adult orthodontic patients. Statistics Netherlands reports that about 5% of all adults in the Netherlands has visited an orthodontic clinic each year between 2014 and 2020, which translates to a near equal number of adolescent and adult patients visiting orthodontic clinics each year [5]. Patients demand more aesthetic forms of orthodontic treatment [6] and technological advancements have led to innovations in orthodontic appliances. Clear aligner therapy (CAT) has been developed [7–9], during which patient adherence is crucial to ensure sufficient effectiveness of these removable appliances. Also, so-called non-compliance appliances have been developed in the form of implant-supported appliances [10], which are marketed as to not require any patient cooperation.

Given these changes and based on recommendations of previous research [2], the question arises whether the insights and consequences of variations in adherence have changed and, if so, in what areas and to what extent? An answer to this question will be sought through a scoping review, in which the following sub-questions will be distinguished.

- Which definitions are used defining patient adherence in orthodontics?
- What is the effect of the degree of adherence on orthodontic treatment outcomes?
- Which methods of measuring patient adherence are used in orthodontics?
- What is known about the level of adherence and factors to influence adherence during both active orthodontic treatment and the retention phase?

## Materials and methods

### Study design

This scoping review provides an evidence-based overview of the literature available on multiple aspects of patient adherence in orthodontics, during both active treatment as well as during the retention phase. The focus will be on the following topics: defining patient adherence in orthodontics, the effect of patient adherence on orthodontic treatment outcomes, methods of measuring patient adherence in orthodontics, and the degree of patient adherence and factors to influence adherence. The findings of this review may be used to conduct further research in the fields in which knowledge gaps are identified.

### Protocol and registration

The protocol for this scoping review was registered in the Open Science Framework (10.17605/OSF.IO/EC6QD). The design of this review was drafted according to the guidelines of the Preferred Reporting Items for Systematic reviews and Meta-Analysis extension for Scoping Reviews (PRISMA-ScR) [11]. In addition, Arksey and O’Malley’s framework [12] and the Reviewer’s Manual of the Joanna Briggs Institute (JBI) for conducting scoping reviews [13] were consulted.

### Eligibility criteria

The following selection criteria were applied:

1. Studies of all designs with the primary aim of investigating any form of patient adherence in orthodontics, with the exception of case reports and studies investigating adherence in the following patients: those with an intellectual or physical disability that could affect their ability to coincide with their therapist’s recommendations and advice, those with oral cleft and craniofacial conditions, and those treated solely for obstructive sleep apnoea. Patients with oral cleft and craniofacial conditions are excluded because of the higher orthodontic burden for this group of patients [14]. This patient group is generally treated in specialized teams and has a longer duration of treatment [14, 15]. Patients solely treated for obstructive sleep apnoea are excluded because of the difference in treatment need and used appliances for this group of patients. Research in adherence for these groups of patients should therefore be reported in separate reviews.
2. Peer-reviewed manuscripts only.
3. Studies published from 2006 onwards. The Academic Centre for Dentistry Amsterdam (ACTA) has been conducting research in patient adherence in orthodontics up to 2006 [16].
4. Studies published in English only.

### Information sources, search strategy and selection of sources of evidence

The electronic search was performed in the following electronic databases: Embase, PubMed and Web of Science Core Collection (Table 1). The following key terms, including synonyms and subheadings of the MeSH terms, were used: ‘treatment adherence and compliance’ and ‘orthodontics’. The electronic search was performed on 9 February 2023. A filter was applied to search only for studies published from 2006 onwards. Reference lists of relevant studies were also screened, and citations were tracked to identify additional eligible studies. The information sources and key terms were selected in consultation with multiple medical information specialists working at the Vrije Universiteit (VU) Amsterdam medical university library.

**Table 1:** Search strategy

Search strategy
*Databases*
PubMed® (MEDLINE®), Embase®, Web of Science Core Collection™
*Search strategy*
1. “Treatment Adherence and Compliance”[Mesh] OR “adher*“[tiab] OR “comply*“[tiab] OR “complian*“[tiab] (1829648)
2. “Orthodontics”[Mesh] OR “Orthodont*“[tiab] (171584)
3. 1 and 2 (4957)
4. 1 and 2, filter: from 2006 - 2023 (3284)

The results were screened for relevant titles, keywords and abstracts independently by two reviewers (RB and RJ). After identification of relevant studies, full texts were obtained and independently evaluated for eligibility according to the predefined selection criteria by the same reviewers. Any disagreement between the two reviewers during this two-stage screening process was resolved by re-reading the studies concerned. Persisting disagreements were resolved by independent validation by a third reviewer (AB or JB).

### Data items and collection process

A pre-piloted data charting form was used for data extraction. This form was pilot tested using 50 randomly selected studies of the draft search strategy during the development of the review protocol. Data on the following was extracted: study design, primary study objective, study methodology, study population and sample size, outcome measures, and key findings related to the research questions. The data was charted by the same reviewers who selected the sources of evidence (R.B. and R.J.). Any disagreement between the two reviewers was resolved by re-reading of the studies concerned. Persisting disagreements were resolved by independent validation by a third reviewer (A.B. or J.B.).

### Quality appraisal and risk of bias assessment

Quality appraisals and risk of bias assessments are optional when conducting a scoping review [11, 12] and are typically not performed. As the eligible studies were expected to include various study designs and lack reported quantitative outcome measures, no quality appraisal or risk of bias assessment was performed.

### Summary measures and synthesis of the results

This review explores the literature on patient adherence during active orthodontic treatment taking different treatment methods and multiple phases of active treatment into account, as well as during the retention phase. The analysis of the included studies is presented in a narrative form, categorized in topics based partially on recommendations of previous research on adherence in orthodontics [2]. Knowledge gaps identified in the literature are listed.

## Results

**Fig. 1:**
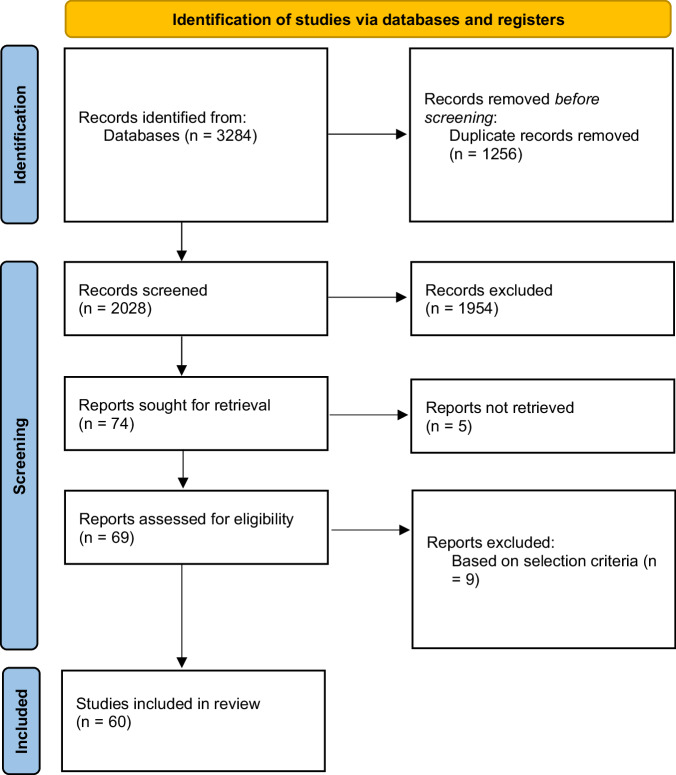
The PRISMA study flow diagram of the studies included in this review. PRISMA study flow diagram.

**Fig. 2:**
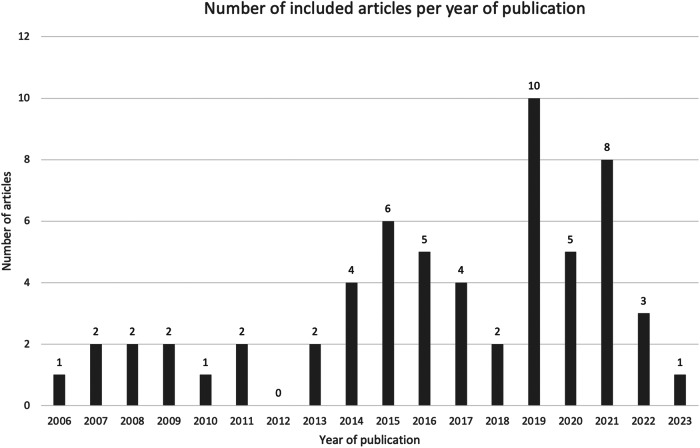
Bar chart showcasing the number of included articles per year of publication. Number of included articles per year of publication.

The electronic search identified a total of 3284 studies. After duplicate removal, 2028 titles and abstracts were screened to identify relevant studies. 74 potentially relevant full-text articles were retrieved and evaluated. 60 articles met the selection criteria for final inclusion (Fig. 1). No additional eligible studies were identified when screening the reference lists of the retrieved articles. The design, primary objective, methodology, population and sample size, outcome measures, and key findings within the included studies are summarized in Supplementary Table 1. The number of included articles per year of publication is shown in Fig. 2.

## Results of individual studies

### The definition of patient adherence in orthodontics

There is no precise definition of patient adherence in orthodontics. Several studies applied Haynes and Dante’s definition of adherence in general healthcare: ‘the extent to which a person’s behaviour coincides with medical or health advice’ [4]. However, most studies used pre-selected outcome measures assessed by the authors as units to measure a specific type of adherence. Because of the variety in applied outcome measures there was little coherence between these studies. The most frequently used outcome measures could be considered as generally accepted key factors to define the degree of adherence. These included: measurable treatment results as decline of overjet, change in occlusion or amount of maxillary expansion, time removable appliances are actually worn compared to the instructed wear time, level of appointment-keeping, level of oral hygiene during treatment, and orthodontic stability during the retention phase.

### Effects of patient adherence on orthodontic treatment outcomes

Studies focusing on the effect of adherence on orthodontic treatment outcomes all investigated the effect of the amount of wear time of removable functional appliances on orthodontic treatment goals. These studies found that for headgear-activators a daily wear time of at least 8 h for at least 5 months was required to achieve significant overjet reduction [17] and that for twin block appliances a daily wear time of at least 8 hours was required to successfully correct class II/1 malocclusions [18]. A study investigating the different treatment outcomes between full-time (>17 hours per day) and part-time (<12 hours per day) twin block wearing groups found significant changes in cephalometric landmarks [19] in favour of the full-time wear group. To achieve sufficient maxillary expansion a daily wear time of at least 9 h of expansion plates was found to be required [20]. The amount of wear time of expansion plates appeared to be associated with the maxillary transverse width increase is shown in another study as well [21].

### Methods of measurement

**Fig. 3:**
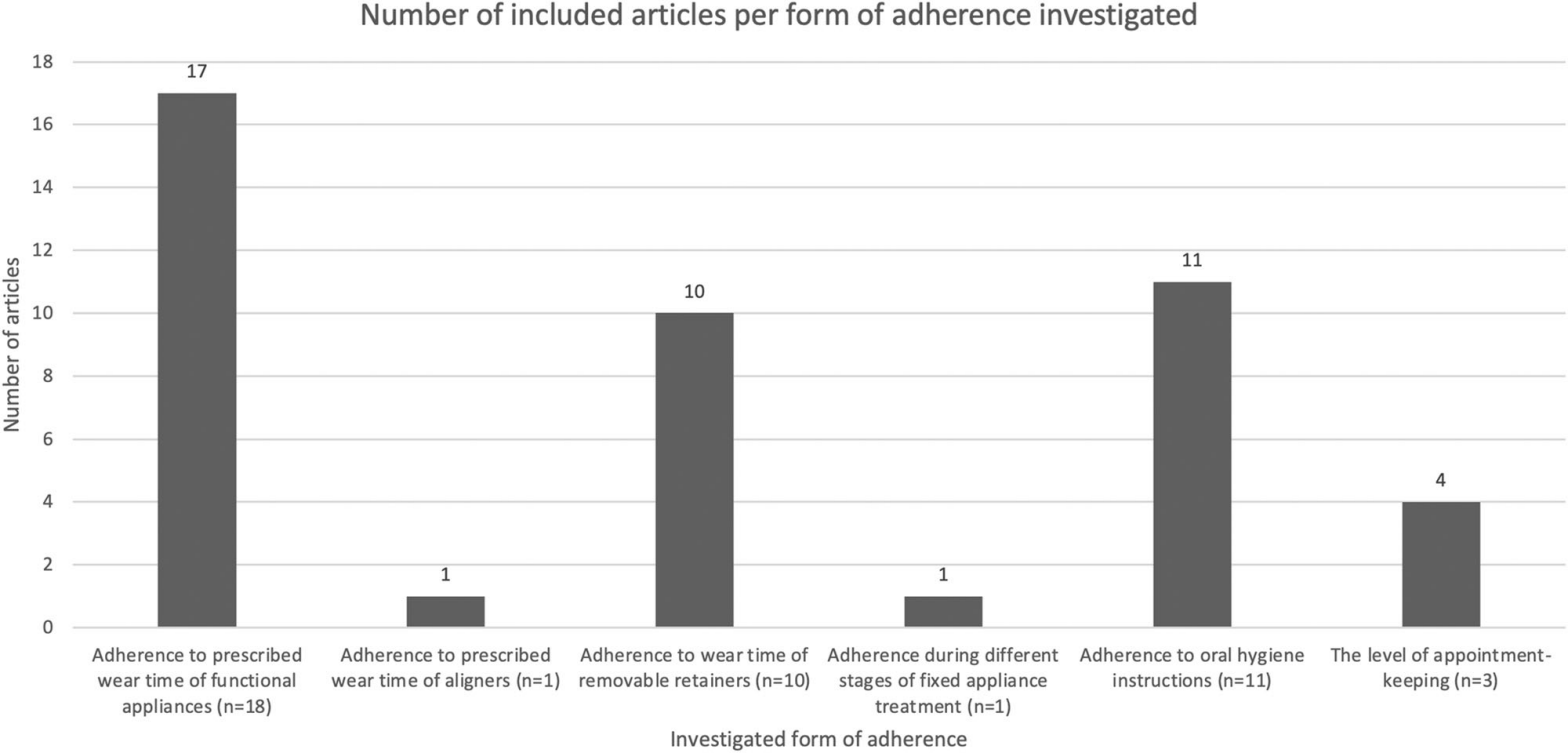
Bar chart showcasing the number of included articles per form of adherence investigated. Number of included articles per form of adherence investigated.

Most of the studies measuring the different selected outcome measures of adherence used only objective measurement methods. Studies investigating adherence to prescribed wear times of removable functional appliances and removable retainers all used temperature-sensitive microsensors to compare the time appliances were actually worn to the instructed wear time [17, 18, 20–34]. Several of these studies combined this sensor data with measured treatment results as overjet reduction, maxillary expansion or change in occlusion [17, 18, 20, 24] to link the degree of adherence to the progression of treatment. To measure appointment-keeping, studies evaluated patient files to compare the number of kept appointments to the number of originally scheduled appointments [35–38]. Studies investigating adherence to oral health instructions during treatment all measured oral hygiene parameters on several moments during the observation period to quantify the degree of adherence to these instructions [39–49]. These parameters included the plaque index, bleeding index, (modified) gingival index, and development of white spot lesions. Several studies used only subjective measurement methods to measure patient adherence. During clear aligner treatment, studies recorded patients’ self-reported daily wear time by the use of an app [50, 51]. To investigate adherence to appointment-keeping, one study used a questionnaire to record self-reported levels of attendance [52]. During the retention phase, studies used questionnaires and interviews to investigate adherence to the prescribed wear times of removable retainers by recording self-reported levels of wear time [53–57]. All studies that used and combined both objective and subjective methods to measure adherence compared the time removable appliances were actually worn to patients’ self-reported wear times and all found that patients consistently overestimate their wear time [58–61]. Figure 3 shows the number of articles investigating the different forms of patient adherence highlighted in this review. Note that several studies were focused on multiple forms or factors of adherence.

### Adherence during active orthodontic treatment

Adherence to the prescribed wear time of functional appliances was investigated in 18 studies. Of these studies, the wear time of headgear appliances was the subject in four studies [25, 32, 58, 59], the wear time of twin block and monobloc appliances was investigated in five studies [18, 24, 27, 28, 60], the wear time of headgear-activators was discussed in one study [17], and the wear time of maxillary expansion appliances in two studies [20, 21]. To investigate the influence of the appliance type on wear time adherence, six studies investigated and compared the wear time of multiple appliances [22, 23, 29, 30, 61, 62]. There was a large discrepancy in the reported mean daily wear time of all removable functional appliances, with a range of 45–[22] 93% [32] of the prescribed daily wear time [17, 18, 20–25, 27–30, 32, 58–62], with no clear difference between the different types of functional appliances. It was reported that up to 92% of patients did not adhere to their prescribed wear time [30]. Regarding adherence to the prescribed wear time of aligners during CAT, one study found that merely 36% of patients adhere to their prescribed wear time [50]. Almost all studies investigating adherence to oral health instructions only reported key findings regarding factors to influence this adherence, but did not provide any significant findings regarding the level of oral hygiene or degree of adherence to these instructions during orthodontic treatment. Only one study investigating the level of adherence to oral hygiene instructions found a decline in oral hygiene with the progression of treatment during different stages of fixed appliance treatment [63]. The studies on the level of appointment-keeping [35–37] reported attendance levels ranging between 67.8% [35] and 91.7% [36]. When assessing the influence of patient demographics, conflicting results were reported about the influence of the patient’s sex, with both males being reported to be more compliant to prescribed wear times of removable appliances and keeping-appointments [36, 50, 59] as females [37, 62], to the patient’s sex having no association with the level of adherence [20]. The patient’s age was reported to have no association with the level of adherence [20]. The effect of the patient’s BMI appeared to be an influencing factor of adherence to prescribed wear times of removable appliances [64] as well as having no relationship to this level of adherence [65]. Studies regarding other patient related factors reported that psychological factors as self-motivation, peer and authority influence, quality of life impairment and adaptability, and perceived treatment progress have a positive effect on adherence [66]. The severity of malocclusion was found to be a predictor of good adherence to wear removable appliances [67], but another study [27] contradicted this finding. It was reported that patients who ranked their smile attractiveness as low showed higher levels of adherence during treatment [68]. It also emerged that patients without a history of previous orthodontic treatment had a higher mean daily wear time of aligners during CAT than patients with a history of previous orthodontic treatment [50]. Studies exploring the effect of a patient’s insurance status appeared to give conflicting results, with insured patients showing a lower level of appointment-keeping and a longer duration of treatment than self-pay patients [38, 52], as well as the type of insurance to have no effect on adherence or treatment duration [69, 70]. One study found that patients with delinquent financial accounts were more likely to miss appointments [37]. Another study showed that the perceived severity of malocclusion, school performance, and parental marital status were identified as positive predictors of adherence to oral hygiene instructions [39]. Multiple studies reported parental involvement to positively affect compliance during active treatment [28, 67, 71]. A high state of anxiety by the patient was found to be a predictor for non-compliance [71]. It was reported that compliance cannot be predicted before treatment by locus of control questionnaires [72]. The effect of reminder messages and mobile applications to enhance adherence to oral hygiene instructions was the subject in several studies. The majority of these studies reported that reminder messages had a positive effect on this adherence [40, 41, 43, 46], however one study [73] reported that reminder messages only improved this adherence if audio-visual material was attached, and one study [47] reported that reminder messages did not have an effect on this adherence. For the use of mobile reminder applications during fixed appliance treatment conflicting findings were reported as well, with both mobile applications that had a positive effect on adherence [44, 49] as no effect at all [42, 48]. The effect of reminder messages was also investigated in other aspects of patient adherence, and it appeared to have a positive effect on treatment outcomes when using intermaxillary elastics [74], in improving the wear time of aligners during CAT [51] and in reducing the number of failed-appointments [45]. Studies on the effect of different types of removable appliances on adherence to the prescribed wear times indicated no difference between headgear-activators, monobloc appliances and twin block appliances [22, 60]. Even when comparing appliances used for class II and class III malocclusion treatment no difference in adherence was found [23]. One study [62] reported less adherence when a headgear was combined to a block appliance and one study [67] reported less adherence when a bite jumping appliance was used compared to a regular twin block appliance. Regarding the effect of the magnitude of orthodontic force applied during treatment, it was found that in a treatment using headgear appliances adherence was better when lighter forces were used compared to heavier forces [32]. Several studies indicated that adherence to prescribed wear times increased after patients were made aware of their wear time being monitored [26, 59], however, one study [61] contradicted this finding. When investigating other treatment related factors to influence the level of adherence, studies found that a change of clinician during treatment had a negative impact on appointment-keeping [35], and that the relationship between patient and orthodontist played a key role in the level of adherence [29]. A more frequent appointment interval did not result in increased adherence to the wear time of removable appliances [24]. One study indicated that the duration of treatment had an effect on adherence by reporting a decline in oral hygiene with the progression of orthodontic treatment [63].

### Adherence during the retention phase

All studies investigating the degree of patient adherence during the orthodontic retention phase looked at the daily wear time of removable orthodontic retainers [26, 31, 33, 34, 53–57, 75]. Five of these studies used temperature-sensitive microsensors to compare the time removable retainers were actually worn to the instructed wear time [26, 31, 33, 34, 75]. These studies found mean daily wear times ranging from 56 to 87.5% [22, 31] of the prescribed daily wear time within three months after completion of orthodontic treatment, which decreased to 38.8% after 12 months of completing orthodontic treatment [34]. The remaining five studies recorded patients’ self-reported levels of retainer wear time and could therefore only provide an estimation of the degree of adherence [53–57]. However, several of these studies also reported a decline in adherence to prescribed retainer wear time as the time after completing orthodontic treatment increased [53, 54]. No difference in wear time adherence was reported between different types of removable retainers, particularly Hawley and vacuum formed retainers [33, 75] even though studies advocated the use of both these types of retainers to enhance adherence [56, 57]. Patients wore their retainers more when they were aware of their wear time being monitored when their retainer was equipped with a microsensor [26]. Parental involvement positively affected adherence to wearing retainers [56]. When patients were shown images illustrating severe consequences of poor adherence this only improved their adherence if their parents were shown these images as well [55]. Mobile reminder applications to wear removable retainers had no effect on adherence to the prescribed wear time of these retainers [34, 75], however one study found that patients participating in a group chat after completing orthodontic treatment showed less orthodontic relapse [76]. The patients’ insurance type was found to have an association with the time retainers are being worn, with privately insured patients being more compliant than patients with statutory health insurance [31]. Patients who were treated at private orthodontic clinics showed a higher mean daily wear time of their retainers when compared to patients treated at university clinics [31, 33].

## Discussion

The aim of this scoping review was to provide an evidence-based overview of the literature available on multiple aspects of patient adherence in orthodontics and identify knowledge gaps. There are no other comprehensive reviews available on patient adherence in orthodontics, only reviews on specific topics of adherence such as the wear time of removable appliances during active treatment [1, 77], methods to improve adherence to oral hygiene instructions [78, 79] and the effect of mobile apps and reminder messages on patient adherence [80–83]. By maintaining a broad research topic it was possible to provide a more comprehensive overview of adherence in orthodontics which created the opportunity to identify knowledge gaps important to every oral healthcare professional practicing orthodontics.

No systematic review has previously assessed this exact topic. Because of the broad-spectrum topic, the likelihood that the literature would be too diverse, and the aim of identifying knowledge gaps, a scoping review approach was chosen over a systematic review approach. The strengths of this scoping review include the broad spectrum of information sources, a research team consisting of topic experts and information scientists, pilot-tested research methods, and peer-reviewed search strategies. Scoping reviews have some limitations compared to systematic reviews, for example registration of the review protocol is not possible in PROSPERO, there is no mandatory risk of bias assessment or critical appraisal, and no quantitative synthesis [84]. We addressed some of these limitations by registering our protocol in the Open Science Framework.

While there is no generally applied definition, patient adherence in orthodontics should be defined as: the extent to which patients follow up on instructions and advice as prescribed by their oral healthcare professional. Key indicators to define the degree of adherence should be the use of orthodontic appliances as instructed, keeping appointments, and maintaining a sufficient level of oral hygiene during treatment. To measure the degree of adherence the use of objective measurement methods is favourable over the use of subjective methods, since the latter significantly overestimate the degree of adherence [58–61]. This is in accordance with the findings of the systematic review by Al-Moghrabi et al. [1]. There is a large discrepancy in reported mean daily wear times of removable functional appliances and attendance levels. A possible explanation is the use of short follow-up periods, usually no longer than six months. Regardless of this it is noticeable that removable appliances are worn less than prescribed, which is in accordance with the findings of the systematic reviews by Al-Moghrabi et al. and Nahajowski et al. [1, 77]. A few studies report minimum required appliance wear times in order to achieve significant treatment results. However, no clear statements about the impact of the degree of adherence on desired treatment results can be provided because most studies do not compare the degree of adherence to achieved treatment results. Only one study investigated the wear time of orthodontic aligners [50] using data of 2644 patients collected by subjective measurement, and found that just 36% of patients adhere to their prescribed daily aligner wear time. The aligner wear protocol is based on a minimum daily wear time [85], so more research to confirm this finding could provide evidence to possibly recommend changes to this protocol. Only one study investigated adherence to oral hygiene instructions during active treatment, even though several studies used oral hygiene parameters as outcome measures for patient adherence. The degree of adherence during different stages of orthodontic treatment was investigated in one single study and more evidence is required to strengthen the statement that adherence decreases with the progression of orthodontic treatment. Even though intermaxillary elastics are commonly used in orthodontics, only one study [74] investigating adherence to the use of these elastics is available. No other reviews on adherence during the retention phase are available. Despite a large discrepancy in reported mean wear times of removable retainers, a decrease in wear time with the progression of time after completing orthodontic treatment is noticeable. Even though many studies attempted to investigate possible factors to influence or promote adherence, conflicting evidence was reported for almost every factor investigated. This makes it impossible to provide clear statements on which factors have a significant impact on adherence and how patient adherence can be promoted. There is no unambiguous evidence about the positive effect of the use of mobile applications, but the majority of studies investigating this subject conclude a positive influence in producing behavioural changes, which is in accordance with the findings of the review of Al-Moghrabi et al. [80]. The findings of our review also agree with the conclusions of the reviews of Patil et al. and Sharif et al. that even though the evidence is weak, mobile applications appear to be helpful in improving adherence to oral hygiene advice and could be recommended to use during orthodontic treatment [82, 83]. This finding that parental influence has a positive influence on adherence during both the active phase as during the retention phase, and that the relationship between patient and orthodontist plays a key role in the level of patient adherence is in accordance with the review of Aljabaa et al. [78], which also advocates to spend time with patients to explain the importance of adherence. The results of the studies included in this review suggest that an increase of treatment duration has a negative impact on adherence during the active treatment phase, as well as the progression of time after completing orthodontic treatment during the retention phase.

The Academic Centre for Dentistry Amsterdam (ACTA) has been conducting research in patient compliance in orthodontics up to 2006 [16]. In this previous research it was concluded that the lack of knowledge about the degree of adherence is partially due to adherence being difficult to measure, that objective measurement methods are favoured over subjective measurement methods, that removable appliances are worn less than prescribed, that the degree of adherence is difficult to predict before the start of orthodontic treatment, that age, sex and type of insurance have no influence on adherence, and that patient satisfaction with orthodontic treatment is mainly determined by the relationship between patient and orthodontist. The findings of our review prove that these conclusions are still valid and clinically relevant 18 years later.

A reasonable amount of research on patient adherence in orthodontics has been conducted over the past 18 years. It appears that only conclusions based on objective measurement methods provide valid evidence, however there is a large discrepancy in reported findings. Also, studies do not relate the degree of patient adherence to treatment results or treatment stability, which could be explained by the use of relatively short examination periods. This applies both to studies investigating the active treatment phase as the retention phase. It is recommended to conduct research with larger study populations followed up over a longer period, or to conduct retrospective research using data of patients who completed comprehensive orthodontic treatment.

It is noteworthy to mention there is no research available if patients are aware of the necessity of adherence prior to starting orthodontic treatment. Also, there is no research investigating how important the orthodontic profession estimates patient adherence on treatment outcomes and treatment stability available. Furthermore, it is essential to investigate and verify the interest in future research in patient adherence within the orthodontic community. Based on the findings of our review it is recommend to conduct future research in the following knowledge gaps:

- The level of importance of patient adherence as rated by the orthodontic community
- Patient awareness of required adherence prior to starting orthodontic treatment
- The effect of the degree of patient adherence on orthodontic treatment results
- Adherence to the recommended wear time of orthodontic aligners
- The degree of adherence during the different stages of orthodontic treatment
- The degree of adherence to the use of intermaxillary elastics
- Which factors have a significant impact on adherence and ways to promote patient adherence, including the use of mobile applications

To justify future research in these topics, the findings of this review could be disseminated to a sample of orthodontists to prioritize research questions for future research.

## Conclusions

There is no conclusive evidence on which factors have a significant impact on patient adherence and how patient adherence can be promoted. The degree of patient adherence is generally not compared to achieved treatment results or stability of treatment results, making it difficult to provide clear statements about the impact of the degree of adherence on desired treatment results or orthodontic stability.

It is recommended to conduct further research using larger study populations followed up over a longer period of time, or by using data of patients who completed comprehensive orthodontic treatment.

### Supplementary information

Supplemental Table 1

## Data Availability

The data that supports the findings of this study are available in the supplementary material of this manuscript.
