# Supplementary material for: Patient adherence in orthodontics: a scoping review
Source: BDJ Open. 2024 Jul 16;10:58. doi: 10.1038/s41405-024-00235-2 (PMC11252357; doi:10.1038/s41405-024-00235-2)
Supplement: Supplementary file 1 — Supplemental Table 1 [file 41405_2024_235_MOESM1_ESM.pdf]

Supplemental Table 1. Summary of the articles included in this review.

Study	Design	Primary objective	Methodology	Population and sample size	Outcome measures	Key findings
(Al-Abdallah et al., 2021)	RCT	Compare the effect of different communication strategies on patient adherence	Subjects were allocated to three groups receiving different communication strategies	108 subjects (age range 12-18 years) treated with fixed appliances	Duration of treatment, appointment-keeping, frequency of appliance breakage	Weekly audio-visual e-mails, treatment of female patients, and patients with a higher level of parents' education reduced appliance breakage (P<0.05). Weekly text messages did not improve adherence. Patients from households with high income had better adherence (P<0.05)
(Al-Jewair et al., 2011)	Observational study	Investigate adherence to oral hygiene instructions in patients treated with fixed appliances	Subjects were provided with oral hygiene instructions before treatment. Their oral hygiene was assessed during a 150-day observational period	41 subjects (age range 12-16 years) treated with fixed appliances	Oral hygiene indices	Perceived severity of malocclusion, school performance, and parental marital status were identified as predictors of good oral hygiene adherence (p<0.05)
(Al-Kurwi et al., 2017)	Observational study	Investigate the association between overjet reduction and wear time of the Van Beek activator appliance	Subjects were instructed to wear a Van Beek activator appliance equipped with a microsensor for 12 hours per day. Wear time was assessed during the first three appointments	28 subjects (mean age 11.6±1.25 years) treated with a Van Beek activator appliance	Objectively measured wear time, overjet reduction in mm	The mean wear time was 7.75±3.66 hours per day. No patient achieved the prescribed wear time of 12 hours per day. Significant overjet reduction was achieved with a daily wear time of at least eight hours for at least five months
(Al-Moghrabi et al., 2019)	Qualitative study	Investigate factors influencing adherence to vacuum-formed retainer wear	Subjects wearing vacuum-formed retainers were interviewed about their level of retainer wear	15 subjects wearing vacuum-formed retainers for at least four years	Self-reported wear time	The high level of reported adherence in the early stages of retention decreased over time due to multiple causes
(Al-Moghrabi et al., 2020)	RCT	Investigate the effect of a mobile application on objectively measured retainer wear time	Subjects wearing thermoplastic retainers were allocated to a group receiving a mobile reminder application and a control group for a three-month observational period	84 subjects wearing thermoplastic retainers up to three months after completing orthodontic treatment	Objectively measured wear time	No significant difference in retainer wear time was found between the two groups
(Arponen et al., 2020)	RCT	Investigate adherence of adolescents treated with headgear activators and twin block appliances	Subjects were allocated to a headgear activator group and a twin block group during a 13-month observational period	52 subjects (mean age 12.6±1.3 years) treated with a headgear activator or twin block appliance	Objectively measured wear time	The mean wear time was 55% less than advised.<br>There was no difference in mean wear time between the different type of appliances
(Arreghini et al., 2017)	Comparative study	Objective assessment of adherence in young patients treated with various types of removable appliances	A group of class II subjects and a group of class III subjects were treated with removable functional appliances equipped with a microsensor during an	14 class II subjects (mean age 9.8 years) and 16 class III subjects (mean age 10.0 years) treated with a removable functional appliance	Objectively measured wear time	The mean wear time was 8.6±2.9 hours a day, 65% of the prescribed 13 hours a day. There was no significant difference in mean wear time

			eight-month observational period			between the class II and class III group
(Baheti et al., 2022)	Comparative study	Investigate the association between objectively measured wear time and treatment efficacy of twin block appliances	Subjects were treated with a twin block appliance equipped with a microsensor. After six months of treatment their objectively measured wear time was compared to changes in cephalometric landmarks	44 subjects (mean age 12.6±0.84 years) treated with a twin block appliance	Objectively measured wear time, change in cephalometric landmarks	Significant changes of several cephalometric landmarks were found between full-time (mean wear time >17 h/d) and poor wear time (mean wear time of < 12 h/d) subjects, with maximum treatment effects occurring in the full-time wear group
(Bos et al., 2007)	Comparative study	Compare subjective and objective methods to measure headgear wear time	Objectively recorded and subjectively reported headgear wear times of subjects were compared	56 subjects (mean age 12.9±2.2 years) treated with a low-pull headgear appliance	Objectively measured wear time, subjective indices of patient adherence	Orthodontists, patients, and parents of patients overestimate the wear time of headgears when compared to the objectively measured wear time ($p<0.05$)
(Bowen et al., 2015)	RCT	Investigate if text message reminders affect oral hygiene in orthodontic patients	Subjects were allocated to a group receiving text messages and a control group for a 12-week observational period	50 subjects treated with fixed appliances (mean age 15.5 years for the test group, 14.6 years for the control group)	Oral hygiene indices	The text message reminder group had less plaque coverage than the control group at all measurement moments ($p<0.05$) and showed a mean reduction in plaque coverage during the observational period ($p<0.05$)
(Brandão et al., 2006)	Comparative study	Compare objectively measured headgear wear times to patient self-reported wear times	Subjects were instructed to wear a headgear appliance for 14 hours per day. Objective and subjective wear time values were collected every two weeks. After 70 days, subjects were made aware of the timing device	21 subjects (age range 11-19 years) treated with a headgear appliance	Objectively measured wear time, self-reported wear time	Patients overestimate their wear time by an average of 8 hours per day. The objectively recorded mean wear time was 56.7% of the prescribed 14 hours a day. This increased to 62.7% after patients were made aware of the microsensor. Males were more compliant than females
(Bukhari et al., 2016)	Qualitative study	Investigate predicting factors to influence attendance at orthodontic appointments	Subjects completed self-administered questionnaires guided by face-to-face interviews	153 subjects (mean age 14.7±3.9 years) with a mean average active treatment time of 21±16 months	Appointment-keeping, duration of treatment	Factors associated with patient adherence to orthodontic appointments include: insurance type, oral hygiene practices, and treatment duration ($p<0.05$)
(Charavet et al., 2019)	Observational study	Investigate patient compliance with removable functional expansion appliances	Subjects were instructed to wear a removable appliance equipped with a microsensor for 24 hours per day during a nine-month observational period. Wear time data was collected every three months	69 subjects (mean age 7.8±1.1 years) treated with a removable expansion appliance	Objectively measured wear time, maxillary transverse expansion in mm	Patients wore the appliance on average 15.8 hours per day (65.8%) instead of the 24 hours prescribed. Sufficient maxillary expansion was achieved with a wear time of at least 9 hours per day. Wear time was unrelated to gender or age

(Cozzani et al., 2016)	RCT	Investigate the effect of follow-up communication on oral hygiene adherence after fixed appliance placement	Subjects were allocated to three groups with different follow-up communication after fixed appliance placement: text-message, phone, and control for an observational period of 30-40 days	84 subjects (mean age text-message group 12.8±1.5 years, mean age phone group 13.6±1.7 years, mean age control group 13.5±1.7 years) treated with fixed appliances	Oral hygiene indices	Subjects in the groups that received follow-up communication on oral hygiene had a lower mean plaque index ($p<0.05$) than the control group. There was no difference between the two test groups
(Deleuse et al., 2020)	RCT	Investigate if the use of a mobile application connected to a toothbrush improves the oral hygiene adherence of adolescent orthodontic patients	Subjects were allocated to a test group using a toothbrush connected to a mobile application and a control group using a toothbrush alone for an observational period of 18 weeks. Data was collected every six weeks	38 subjects (mean age 13.9±1.2 years) treated with fixed appliances	Oral hygiene indices	The use of a mobile application connected to a toothbrush had no clinically relevant effect on the oral hygiene of orthodontic patients
(Dickens et al., 2008)	Comparative study	Investigate if there is a difference in patient adherence and treatment outcomes between Medicaid and non-Medicaid orthodontic patients	Records of Medicaid and non-Medicaid cases that completed orthodontic treatment at private practices were evaluated	Orthodontic records of 43 Medicaid cases and 42 non-Medicaid cases that completed orthodontic treatment	Appointment-keeping, frequency of appliance breakage, oral hygiene indices, Peer Assessment Rating index	No difference between Medicaid and non-Medicaid patients regarding patient adherence, treatment duration, and treatment outcomes has been found
(Dobbs et al., 2015)	Comparative study	Investigate if there is a difference in patient adherence between Medicaid and non-Medicaid orthodontic patients	Records of Medicaid and non-Medicaid cases in active comprehensive orthodontic treatment at both university and private practices were evaluated	Orthodontic records of 30 Medicaid cases and 30 non-Medicaid cases that completed orthodontic treatment	Appointment-keeping, frequency of appliance breakage, oral hygiene indices	No difference between Medicaid and non-Medicaid patients in adherence was found
(El-Huni et al., 2019)	Observational study	Investigate factors influencing adherence in adolescents treated with a twin block appliance	Subjects treated with a twin block appliance equipped with a microsensor were interviewed about their level of adherence	22 subjects (mean age 12.5 years) treated with a twin block appliance for ≥3 months	Objectively measured wear time	Factors found to influence adherence include: self-motivation, peer and authority influence, quality of life impairment and adaptability, perceived treatment progress, and pragmatic and recall issues. Recommendations to improve adherence included: effective communication, tailoring of prescribed wear duration, physical alteration of the appliance, and use of reminding tools
(Eppright et al., 2014)	RCT	Investigate if text-message reminders influence oral hygiene adherence in fixed appliance treatment	Subjects treated with fixed appliances were allocated to a text message group and a control group during an observational period of four appointments	42 subjects (age range 11-18 years) treated with fixed appliances	Oral hygiene indices	Bleeding index, modified gingival index, and plaque index were lower when patients received text-messages regarding oral hygiene adherence ($p<0.05$)
(Farhadifard et al., 2020)	RCT	Investigate the efficacy of a smartphone application for oral	Subjects treated with fixed appliances were assigned to a group using a mobile	120 subjects (mean age test group 18.7±3.8 years, mean age	Oral hygiene indices	Patients who used the oral hygiene compliance app showed a lower plaque index

		hygiene adherence of patients treated with fixed appliances	application and a control group during a 12-week observational period	control group 19.37±3.7 years) treated with fixed appliances		and gingival index than the control group patients ($p<0.05$). In the test group, the app usage frequency was positively correlated with the brushing duration and frequency
(Fonseca et al., 2018)	Retrospective study	Identify factors associated with patients failing to keep orthodontic treatment appointments	Records of 237 subjects who completed orthodontic treatment collected from multiple clinics were evaluated	Orthodontic records of 237 subjects who completed comprehensive orthodontic treatment	Appointment-keeping	The percentage of appointment keeping for a total of 8283 scheduled appointments was 67.8%. The main factor identified to negatively influence appointment keeping was change of the clinician ($p<0.05$)
(Frilund et al., 2023)	RCT	Compare the effect of different check-up prescriptions on patient adherence and treatment outcomes during treatment with a twin block appliance	Subjects treated with a twin block appliance equipped with a microsensor were allocated to a group with a four-week appointment interval and a group with a six-week appointment interval. Changes in overjet, overbite and molar relationship were assessed on study casts after completing treatment	73 subjects (mean age four-week interval group 11.6±1.9 years, mean age six-week interval group 10.9±1.4 years) treated with a twin block appliance	Objectively measured wear time, change in overjet, overbite, or molar relationship	A more frequent check-up interval did not result in increased wear-time and/or improved treatment results in patients treated with a twin block appliance. The mean wear-time was 6.5 hours per day, 54.2% of the prescribed 13 hours a day
(Horsley et al., 2007)	Comparative study	Investigate if there is a difference in appointment-keeping between Medicaid and non-Medicaid orthodontic patients	Appointment-keeping was evaluated for 185 Medicaid and 522 non-Medicaid subjects in active orthodontic treatment at a university clinic during a 12-month observational period	Appointment-keeping records of 707 subjects in active orthodontic treatment at a university clinic	Appointment-keeping	Medicaid patients failed 15.4% of 1609 appointments, non-Medicaid patients failed 8.3% of 4438 appointments ($p<0.05$). Across-group differences showed that females had a higher failure rate than males ($P<.05$)
(Huanca Ghislanzoni et al., 2019)	Observational study	Investigate headgear wear time	Subjects were treated with a headgear appliance equipped with a microsensor. Wear time was measured during an eight-month observational period	20 subjects (mean age 12.1±1.2 years) treated with a cervical headgear appliance	Objectively measured wear time	The appliance was on average worn at least once per day on 70% of the observed days. Mean wear time was 8.7 hours (73%) of the prescribed 12 hours a day on days the appliance was worn
(Hyun et al., 2015)	Comparative study	Evaluate the adherence of patients wearing Hawley retainers	Subjects instructed to wear a Hawley retainer equipped with a microsensor were allocated to a group informed about the presence of the microsensor and a group not informed about the presence of the microsensor for a 12-week observational period	22 subjects (mean age 15.6±1.3 years) instructed to wear a Hawley retainer	Objectively measured wear time	Patients made aware of the monitoring device wore the appliance more than the patients not made aware ($p<0.05$). The average wear times were 86% for the test group and 56% for the control group of the prescribed wear time of 19 hours a day

(Kacer et al., 2010)	Qualitative study	Investigate retainer wear in the first two years after completing orthodontic treatment	Subjects completed questionnaires on their prescribed retention protocol adherence	428 subjects instructed to wear a removable retainer	Self-reported wear time	Daily night-time wear rates decreased from 69-76% at six months after debond to 45% at 24 months after debond. Age, sex, and type of retainer did not influence the levels of adherence. 81% of the subjects wore their retainer at least one night per week at 24 months after debond
(Kutay et al., 2021)	Comparative study	Investigate objective adherence levels in patients wearing monobloc and twin block functional appliances	Subjects were allocated to a monobloc group and a twin block group and instructed to wear their appliance 15 hours per day. Wear time was measured objectively using a microsensor and subjectively using wear time charts during an observational period of six appointments	30 subjects (mean age monobloc group 12.7±1.4 years, mean age twin block group 12.3±1.0 years) treated with a monobloc or twin block appliance	Objectively measured wear time, self-reported wear time	The mean objectively recorded wear time was 10.7±3.9 hours, 71.3% of the prescribed wear time. There was no difference between the two appliances. Patients reported more wear time by an average of 3.8 hours
(Lee et al., 2008)	Observational study	Investigate the association between orthodontic patient compliance and locus of control (LOC) characteristics and evaluate post-treatment changes in LOC profiles	Subjects completed two types of LOC questionnaires before and after orthodontic treatment	561 subjects (mean age 16.4±6.5 years) who completed comprehensive orthodontic treatment	Appointment-keeping, oral hygiene indices	Orthodontic patient adherence cannot be predicted before treatment by LOC questionnaires
(Leone et al., 2019)	RCT	Investigate the influence of text messages on cooperation of Class II patients using intermaxillary elastics	Subjects using intermaxillary elastics were allocated to a group receiving text messages twice a week and a control group during a three-month observational period	42 subjects (mean age test group 18.4±6.2 years, mean age control group 19.7±6.8 year) treated with fixed appliances using intermaxillary elastics	Change in sagittal landmarks	Patients who received text message reminders showed a 3.7 times greater correction than patients who did not receive reminders ($p<0.05$)
(Li et al., 2016)	RCT	Investigate the effect of a messaging app in improving adherence and reducing treatment duration	Subjects treated with fixed appliances were allocated to a group receiving a reminder messaging app and a control group and were observed until completion of treatment	224 subjects (mean age 17.6±5.7 years) treated with fixed appliances	Duration of treatment, appointment-keeping, frequency of appliance breakage, oral hygiene indices	Patients who received reminder messages during treatment showed less failed appointments, bracket bond failures, and had a shorter duration of treatment ($p<0.05$). The number of failed appointments was identified as an independent factor affecting duration of treatment
(Lin et al., 2015)	RCT	Investigate if verbal instructions combined with images showing severe consequences of poor adherence can improve retainer wear	Subjects instructed to wear retainers were allocated to two test groups shown images illustrating consequences of poor retention compliance and a control group during a	302 subjects (age range 11-18 years) instructed to wear a Hawley retainer	Self-reported wear time	Patients shown images illustrating severe consequences of poor retention compliance only had a greater daily wear time when their parents were shown the images as well ($p<0.05$)

			three-month observational period			
(Lindauer et al., 2009)	Retrospective study	Investigate the effect of the patient's financial status on orthodontic appointment attendance	Appointment-keeping of 538 scheduled appointments of non-Medicaid subjects was evaluated and compared to patients' financial statuses	538 scheduled appointments of non-Medicaid subjects treated at a university clinic	Appointment-keeping	87.7% of the scheduled appointments were kept. Patients with delinquent financial accounts are more likely to miss appointments ($p<0.05$). Females kept more appointments than males ($p<0.05$)
(Mirzakouchaki et al., 2016)	Comparative study	Investigate the association between various clinical and social factors and adolescent orthodontic patients' retention compliance	Subjects who finished orthodontic treatment completed a questionnaire about their retention protocol adherence of the last three months	77 subjects (age range 11-17 years) instructed to wear Hawley or vacuum formed retainers (VFRs)	Self-reported wear time	Daily wear time was greater VFRs than in Hawley retainers ($p<0.05$). Parental attitude and doctor-patient relationship had an impact on daily wear time ($p<0.05$)
(Nahajowski et al., 2022)	Comparative study	Investigate the degree of patient adherence when using different types of removable appliances	Subjects were treated with different types of removable appliances (based on their malocclusion) equipped with a microsensor during a six-month interval period	167 subjects (mean age 10.3 years) treated with either a block appliance, Schwarz appliance, or block appliance in combination with headgear	Objectively measured wear time	Mean daily wear time of block appliances was longer compared to all both other appliances ($p<0.05$). Mean daily wear time was longer in female than male subjects ($p<0.05$)
(Pauls et al., 2013)	Comparative study	Investigate the effect of wear-time recording awareness on appliance wear time	Subjects treated with a removable appliance equipped with a microsensor were allocated to a group made aware of the sensor and a control group not made aware until the first appointment after placement	32 subjects (mean age 12.8±3.7 years) treated with a removable appliance	Objectively measured wear time, self-reported wear time	Mean daily wear time was 54-56% of the prescribed 15 hours. Patients overestimate their daily wear time by a mean of 2.7 hours ($p<0.05$). Awareness of a wear time measuring device does not affect wear time of removable appliances
(Pratt et al., 2011)	Qualitative study	Investigate and quantify orthodontic retainer wear and identify predictors of adherence with removable retainers	Subjects instructed to wear removable retainers completed a questionnaire about their retention adherence within the past six years	280 subjects (age range 8-72 years) who completed orthodontic treatment with fixed appliances within the past six years and were instructed to wear a removable retainer	Self-reported wear time	Adherence is initially greater with vacuum formed retainers but is greater overall with Hawley retainers. Understanding of proper retainer compliance, time since debond, age, sex, and type of retainer were found to be determinant factors for retention adherence ($p<0.05$). Aesthetic concerns were not a significant determinant of retention adherence
(Qabool et al., 2020)	Comparative study	Investigate adherence in adult orthodontic patients during levelling and alignment, working stage and finishing stage of orthodontic treatment	Adherence of subjects in different stages of fixed appliance treatment was assessed and compared using the Patient Cooperation Scale (OPCS)	114 adult subjects (mean age 25.4±6.3 years) treated with fixed appliances	Patient Cooperation Scale (OPSC), Clinical Compliance Evaluation (CCE)	There is a decline in oral hygiene with the progression of orthodontic treatment ($p<0.05$). CCE scores showed a decline in adherence after the working stage ($p<0.05$). There was no difference in OPCS

			and Clinical Compliance Evaluation (CCE) forms			among different stages of treatment
(Ross et al., 2019)	RCT	Investigate if daily text messages improve oral hygiene more than weekly text messages in adolescent orthodontic patients	Subjects were allocated to a group receiving daily text messages and a group receiving weekly text messages. Oral hygiene was assessed at the beginning of the study and again 8.6±0.9 weeks later	79 subjects (age range 12-17 years) treated with fixed appliances at a university clinic	Oral hygiene indices	Patients receiving daily compared to weekly reminders show a lower plaque index, bleeding index and gingival index (p<0.05)
(Sarul et al., 2019)	Comparative study	Investigate if smile attractiveness and its importance are reliable predictors of patient adherence during orthodontic treatment with removable appliances	Before treatment with a removable appliance equipped with a microsensor, subjects and their caregivers completed questionnaires ranking both investigated variables. After nine months, the results were compared to the patient's daily appliance wear time	97 subjects (age range 9-12 years) treated with removable appliances	Objectively measured wear time	Patients who rank to their smile attractiveness as low show a higher daily mean wear time (p<0.05)
(Sarul, Kawala, et al., 2017)	Comparative study	Investigate if orthodontic treatment needs affect patient adherence	Subjects treated with a removable appliance equipped with a microsensor were divided into four groups according to their Index of Orthodontic Treatment Need (IOTN) score. Wear time data was assessed every six weeks during a nine-month observational period	58 subjects (age range 9-12 years) treated with a removable appliance	Objectively measured wear time	The severity of malocclusion has little influence on the level of patient adherence regarding daily wear time of removable appliances. Overall, patients wear their appliance 54.1-75.7% of the prescribed daily wear time
(Sarul, Lew, et al., 2017)	Observational study	Investigate the effect of personality traits of patients and their caregivers on their adherence to wear removable appliances	Personality traits of subjects and their caregivers were assessed at the start of orthodontic treatment with removable appliances equipped with a microsensor. Wear time assessed every six weeks during a nine-month observational period	38 subjects (age range 9-12 years) treated with a removable appliance	Objectively measured wear time	The mean daily wear time was 50.1% of the prescribed daily wear time. Emotionality of the child affected wear time negatively (p<0.05). The severity of the requirements imposed on the subject, a sense of self-efficacy and conscientiousness of the caregivers affected wear time positively (p<0.05)
(Sarul et al., 2021)	Observational study	Investigate the effect of the amount of daily wear time of removable appliances on treatment outcomes during class II malocclusion treatment	Subjects were treated with a twin block appliance equipped with a microsensor. Pre- and post-treatment changes in	55 subjects (mean age 10.4 years) diagnosed with a class II/1 malocclusion and treated with a twin block appliance	Objectively measured wear time, change in clinical parameters, change in cephalometric landmarks	The mean daily wear time was 7.6 hours (54.3-63.3%) of the prescribed 12-14 hours a day. To correct class II/1 malocclusions a daily wear

			clinical and cephalometric parameters were compared to the daily wear time after completion of active orthodontic treatment			time of at least 8 hours is required
(Saxena & Gunjal, 2021)	RCT	Investigate the effect of electronic message reminders on oral hygiene adherence of orthodontic patients	Subjects were assigned to two groups receiving different types of electronic reminders and a control group. Plaque measurement took place after four and eight weeks	54 subjects (age range 18-27 years) treated with fixed appliances at a university clinic	Oral hygiene indices	Electronic reminder messages did not significantly affect oral hygiene adherence.
(Schäfer et al., 2015)	Observational study	Quantify the wear times of removable appliances during active orthodontic treatment	Subjects were treated with a removable appliance equipped with a microsensor. Wear time data was collected every check-up appointment during a three-month observational period	141 subjects (mean age 11.0±1.9 years) treated with a removable appliance	Objectively measured wear time	The mean daily wear time was 9.7 hours (64.7%) of the prescribed 15 hours a day. The relationship between orthodontist and patient seems to play a key role in patient adherence
(Schott et al., 2014)	Observational study	Investigate the influence of screw activation rates and electronically tracked wear times on transverse maxillary expansion with removable orthodontic appliances	Subjects were instructed to wear a removable maxillary expansion appliance equipped with a microsensor for 15 hours per day. Wear time data and maxillary screw gap width was assessed every follow-up appointment during five- to seven-month observational period	28 subjects (mean age 10.6±2.2 years) treated with a maxillary expansion removable appliance	Objectively measured wear time, maxillary transverse expansion in mm	Mean daily wear time was 12.8 hours (85,3%) of the prescribed 15 hours a day.<br>Daily wear time was positively associated with maxillary transverse gap width increase
(Schott & Ludwig, 2014a)	Observational study	Investigate if microelectronic wear-time documentation can contribute to individualized orthodontic management	Wear time and behaviour of subjects treated with a removable appliance equipped with a microsensor were quantified and analysed during a six-month observational period	281 subjects (mean age 11.6±2.4 years) treated with a removable appliance at a private clinic	Objectively measured wear time	The mean daily wear time was 9.0 hours (60-75%) of the prescribed 12-15 hours a day. 92% of the patients did not adhere to the prescribed daily wear time
(Schott & Ludwig, 2014b)	Comparative study	Quantify the association between BMI and wear time of removable orthodontic appliances and evaluate BMI changes during orthodontic treatment	Wear time of subjects treated with a removable appliance equipped with a microsensor was assessed and compared to the subjects' BMI values during a five-month observational period	92 subjects, of which 53 normal weight (mean age 11.2±2.2 years) and 37 overweight (mean age 11.6±2.6 years), treated with a removable appliance	Objectively measured wear time	No statistically significant differences in usage or adherence between normal weight and overweight patients were found

(Schott et al., 2013)	Observational study	Quantify removable retainer wear time during the retention phase using a microsensor	Subjects were instructed to wear a removable retainer equipped with a microsensor at least 8 hours per day. Wear time data was collected during a 90-day observational period	100 subjects (age range 13-20 years) wearing a removable retainer	Objectively measured wear time	The mean daily wear time was 7.0 hours (87.5%) of the prescribed 8 hours a day. Place of treatment and health insurance status influenced adherence more than age or sex
(Scribante et al., 2021)	RCT	Investigate the effect of a mobile application in improving oral hygiene adherence and knowledge in young orthodontic patients compared to traditional chairside verbal instructions	Subjects were allocated to a group receiving chairside verbal oral hygiene instructions at the start of their treatment and a group receiving additional weekly multimedia contents during a six-month observational period	40 subjects (mean age 16.2±1.5 years) treated with fixed appliances	Oral hygiene indices	There was no difference in oral hygiene between patients who received verbal instructions combined with weekly sent multimedia contents and patients who received verbal instructions only
(Stefanovic et al., 2021)	Observational study	Investigate predicting factors of patient adherence during functional treatment of Class II/1 malocclusion patients	Subjects were instructed to wear a removable functional appliance during a one-year observational period. Before treatment, patients and their parents independently filled out multiple questionnaires assessing possible predictors of patient adherence. Overjet reduction in mm after one year was compared to the questionnaire data	77 subjects (mean age 12.0±0.9 years) with a Class II/1 malocclusion treated with a removable functional appliance	Overjet reduction in mm	Parental perception of the child's emotional well-being alteration, severity of malocclusion, and type of appliance were found to be predictors of patient adherence ($P<0.05$)
(Talvitie et al., 2019)	Comparative study	Investigate the effect of applied force during orthodontic treatment with headgear appliances on patient adherence	Subjects were allocated to a light force (300 grams) and heavy force (500 grams) group, and were instructed to wear a cervical headgear appliance equipped with a microsensor for 10 hours per day during a ten-month observational period	40 subjects (mean age light force group 9.7±0.7 years, mean age heavy force group 9.9±0.7 years) treated with a cervical headgear appliance	Objectively measured wear time	Patients in the light force group had a higher daily wear time than patients in the heavy force group ($p<0.05$). Mean daily wear time of the light and heavy force groups were 9.3 and 7.7 hours, respectively (77-93% of the prescribed wear time)
(Timm et al., 2021)	Observational study	Investigate adherence and associated factors in patients treated with clear aligner therapy (CAT)	Subjects were instructed to wear orthodontic aligners ≥22 hours per day and report their daily wear time using a mobile application. Wear time data was analysed after completing treatment	2644 subjects (age range 18-64 years) who completed orthodontic treatment with clear aligners	Self-reported wear time	36% of patients wore their aligners ≥22 hours on ≥75% of their aligner treatment. Male patients had a higher mean daily wear time than female patients ($p<0.05$), patients without previous orthodontic treatment had a higher mean daily wear time than patients with previous orthodontic treatment ($p<0.05$)

(Timm et al., 2022)	Comparative study	Investigate the effect of remote electronic reminders and feedback on adherence during clear aligner therapy (CAT)	Subjects' self-reported wear time data was compared between an intervention group completing CAT while receiving electronic reminders and feedback and a control group completing CAT without receiving electronic reminders and feedback	7385 subjects who finished CAT. 1899 control group subjects (mean age 28.8±7.4 years), 5486 intervention group subjects (mean age 29.1±8.1 years)	Self-reported wear time	Self-reported daily appliance wear time was higher in the group that received electronic reminders and feedback during treatment than in the control group ($p<0.05$)
(Trakyali et al., 2009)	Comparative study	Investigate the effect of parents' attitude, patient's anxiety during treatment, and patient's self-confidence/self-care on patient cooperation during orthodontic treatment	After 18 months of active orthodontic treatment, subjects and their parents were divided into a compliant and non-compliant group based on their oral hygiene during treatment, appointment-keeping, and appliance handling. Both groups completed multiple psychological questionnaires	82 subjects (mean age 15.5±3.3 years) with both parents in their household, undergoing treatment with fixed appliances at a university clinic	Oral hygiene indices, appointment-keeping, level of appliance handling, psychological questionnaire scores	Non-compliant patients revealed a high state anxiety level. Authoritative attitude of the father is found to be dominant at the level of state anxiety of the child
(Vagdouti et al., 2019)	RCT	Objectively investigate patient compliance with Hawley or vacuum-formed retainers during a three-month period after completing orthodontic treatment	Subjects were instructed to wear a retainer equipped with a microsensor and were allocated to a Hawley group and a vacuum-formed retainer group. Wear time data was assessed during a three-month observational period	77 subjects (age range 12-17 years) wearing a removable retainer after completing comprehensive orthodontic treatment with fixed appliances	Objectively measured wear time	Mean daily wear time for the Hawley group was 18.3 hours (76.3% of the prescribed 24 hours), mean daily wear time for the vacuum-formed group was 15.3 hours (63.8% of the prescribed 24 hours). Patients treated at private orthodontic practices showed a higher mean daily wear time compared to patients treated at university clinics ($p<0.05$)
(von Bremen et al., 2018)	Comparative study	Investigate if childhood overweight is associated with lower levels of compliance during orthodontic therapy with removable appliances	Subjects were treated with removable appliances equipped with a microsensor during a six-month observational period. Pre-treatment BMI values were used to divide subjects into a normal weight group and an overweight group	114 subjects of which 57 normal weight and 57 overweight treated with a removable appliance	Objectively measured wear time	An increased BMI appears to be a risk factor for less appliance wear during orthodontic treatment with removable appliances ($p<0.05$)
(Wilson & Harris, 2015)	Comparative study	Investigate differences in adherence between self-pay and Medicaid- supported patients	Records of Medicaid and self-pay subjects who completed orthodontic treatment were evaluated	Orthodontic records of 88 Medicaid subjects and 145 self-pay subjects who completed orthodontic treatment	Appointment-keeping, frequency of appliance breakage, duration of treatment	Medicaid patients showed a lower level of appointment keeping and a longer duration of treatment ($p<0.05$). No difference in the level of appliance breakage was found

(Zhang et al., 2021)	RCT	Investigate the effect of a mobile application on adherence with removable retention	Subjects wearing a removable retainer equipped with a microsensor were allocated to a group given access to a bespoke mobile application providing retention reminders and a control group. Wear time data was collected every three months after completing orthodontic treatment during a 12-month observational period	84 subjects (mean age test group 17.2±2.0 years, mean age control group 17.2±1.9 years) wearing thermoplastic retainers after completing comprehensive orthodontic treatment with fixed appliances	Objectively measured wear time, stability of orthodontic treatment outcome	The mean daily wear time after 12 months was 3.1 hours a day (38.8%) of the prescribed eight hours a day. The use of the mobile reminder application did not improve the daily wear time. There was no difference in orthodontic stability between the two groups
(Zotti et al., 2016)	RCT	Investigate the effect of an app-based approach on oral hygiene adherence in patients treated with fixed appliances	Subjects treated with fixed appliances were allocated to a group receiving oral hygiene instructions and participating in a chat group sharing pictures of their oral hygiene and a control group receiving oral hygiene instructions only. Oral hygiene was assessed every three months during a 12-month observational period	80 subjects (mean age test group 14.1 years, mean age control group 13.6 years) treated with fixed appliances	Oral hygiene indices	Patients participating in a group chat during orthodontic treatment showed a lower plaque index, gingival index, and less white spot lesions ($p<0.05$)
(Zotti et al., 2019)	Comparative study	Investigate if the use of social media is useful in improving adherence and follow-up attendance among patients wearing retainers after orthodontic treatment	Subjects wearing removable retainers were allocated to a group participating in a chat group sharing pictures of their occlusion and a control group. Treatment stability was assessed every four months after completing orthodontic treatment during a 12-month observational period	60 subjects (age range 16-19 years) wearing removable retainers after completing comprehensive orthodontic treatment with fixed appliances	Stability of orthodontic treatment outcome	Patients participating in a group chat after completing orthodontic treatment showed less orthodontic relapse ($p<0.05$)
